# Construction of an Electron Transfer Mediator Pathway for Bioelectrosynthesis by *Escherichia coli*

**DOI:** 10.3389/fbioe.2020.590667

**Published:** 2020-10-15

**Authors:** Jiao Feng, Qiuhao Lu, Kang Li, Sheng Xu, Xin Wang, Kequan Chen, Pingkai Ouyang

**Affiliations:** State Key Laboratory of Materials-Oriented Chemical Engineering, College of Biotechnology and Pharmaceutical Engineering, Nanjing Tech University, Nanjing, China

**Keywords:** electron transfer mediator, phenazine-1-carboxylic acid, *Escherichia coli*, bioelectrocatalysis, succinate electrosynthesis

## Abstract

Microbial electrosynthesis (MES) or electro-fermentation (EF) is a promising microbial electrochemical technology for the synthesis of valuable chemicals or high-value fuels with aid of microbial cells as catalysts. By introducing electrical energy (current), fermentation environments can be altered or controlled in which the microbial cells are affected. The key role for electrical energy is to supply electrons to microbial metabolism. To realize electricity utility, a process termed inward extracellular electron transfer (EET) is necessary, and its efficiency is crucial to bioelectrochemical systems. The use of electron mediators was one of the main ways to realize electron transfer and improve EET efficiency. To break through some limitation of exogenous electron mediators, we introduced the phenazine-1-carboxylic acid (PCA) pathway from *Pseudomonas aeruginosa* PAO1 into *Escherichia coli*. The engineered *E. coli* facilitated reduction of fumarate by using PCA as endogenous electron mediator driven by electricity. Furthermore, the heterologously expressed PCA pathway in *E. coli* led to better EET efficiency and a strong metabolic shift to greater production of reduced metabolites, but lower biomass in the system. Then, we found that synthesis of adenosine triphosphate (ATP), as the “energy currency” in metabolism, was also affected. The reduction of menaquinon was demonstrated as one of the key reactions in self-excreted PCA-mediated succinate electrosynthesis. This study demonstrates the feasibility of electron transfer between the electrode and *E. coli* cells using heterologous self-excreted PCA as an electron transfer mediator in a bioelectrochemical system and lays a foundation for subsequent optimization.

## Introduction

Microbial electrosynthesis (MES) or electro-fermentation (EF) relies on microbial cells as catalysts to increase terminal production of high value fuels and chemicals via electricity (Schroder et al., 2015). They provide a promising strategy for the conversion of electrical energy (current) to chemical energy (extracellular multi-carbon products) to achieve energy storage and distribution and therefore have been widespread concerned. Initially, ancetate was produced from the reduction of CO_2_ with the help of MES (Nevin et al., 2010). Among the developing microbial electrochemical technologies currently known, MES and EF have widespread applications in optimization of more high-value terminal metabolite production from CO_2_ or other substrates (Wang and Ren, 2013; Ganigue et al., 2015; Wu et al., 2019). In bioelectrochemical reactors, microbial cells grow and catalyze reactions at high overpotentials (negative cathode potential) during MES or EF operation. Driven by an electrical current, microbial cells interact with an electrode through a series of oxidation/reduction reactions and electron transfer reactions to stimulate and change the substantial metabolism, energy metabolism or microbial growth (Rosenbaum et al., 2011). It is also able to generate intracellular reducing equivalents by using the electrons, which offers a possible route to break through the limitation of metabolic redox state/balance and energy carriers (Harrington et al., 2015a). Therefore, the primary task is to create cell-electrode connections with inward EET pathway and crucial to MES is the ability and efficiency of microbes to perform the EET process of electron transfer from extracellular redox-active electron donors to intracellular acceptors.

An often-used and attractive method to realize electron flow from extracellular electrodes into microorganisms is the use of electron mediators in bioelectrochemical systems. The electron mediators could be used by both suspended microorganisms in the medium and immobilized microorganisms at the electrode (Patil et al., 2012). Among the electron mediators employed, artificial exogenous mediators like neutral red (Harrington et al., 2015a), methyl viologen (Aulenta et al., 2007), and 2,4-anthraquinonedisulfate (Thrash et al., 2007) have been successfully used in bioelectrochemical systems to yield more valuable fuels and reduced chemical or treat wastewater, and perform well in electron transfer. However, artificial exogenous mediators also have some disadvantages, such as exhibiting limited stability, having short lifespans, being costly to implement, and their uncertainty of sustainability in large-scale applications (Patil et al., 2012). Thus, developing cost-efficient and sustainable electron mediator that does not require continuous addition is one of major challenges. In contrast to this method of exogenous addition of artificial electron mediators, self-secreted electron mediators derived from microorganisms could overcome this challenge.

The currently reported self-secreted electron mediators are mostly produced and secreted by electrochemically active bacteria (EAB) to facilitate electron transfer from the cathode. Examples include pyrroloquinoline quinone secreted by *Acinetobacter calcoaceticus* NBRC12552 (Freguia et al., 2010) and self-secreted flavins by *Shewanella loihica* PV-4 (Liu et al., 2012). Although direct electron transfer from cathodes to microorganisms has been attributed to the Mtr pathway in *Shewanella oneidensis* MR-1, increased production of electron mediators (flavins) have been shown to promote an inward current (Yang et al., 2015). It also has been reported that mediated electron transfer is most important mode of EET. While EAB has excellent ability of self-secreted electron mediators, further application on EAB is limited by the narrow spectrum of substrates and simple compounds that can be synthesizes. And their sophisticated molecular biology tools and available synthetic biological toolsets are not mature. Conversely, model microbe like *Escherichia coli* possess many advantages in genetic modifications and chemical biosynthesis.

Here, we heterologously introduced the synthesis pathway of electron mediators into the model microbe, *E. coli*, to exploit the advantages of electron mediators while simultaneously alleviating the limitations of EAB. Phenazine-1-carboxylic acid (PCA) is a type of redox-active phenazine and is produced by species of *Pseudomonas* and *Actinomycetes*. PCA possesses a relatively low for redox potential (*E* = −0.24 V vs. Ag/AgClsat) and is primarily involved in reversible redox cycling under oxygen-limited conditions (Bosire et al., 2016; Bosire and Rosenbaum, 2017). Reportedly, PCA rather than pyocyanin may be responsible for electron transfer from the cathode to *Pseudomonas aeruginosa* PA14 (Bosire and Rosenbaum, 2017). In our previous our work, we established that PCA could perform the role of electron transfer and effectively improve current generation in microbial fuel cell by introducing the PCA pathway of *P. aeruginosa* in *E. coli* (Feng et al., 2018). PCA production remained relatively stable and the concentration of self-excreted PCA increased with the operation of bioelectrochemical reactor (Feng et al., 2018). However, critical metabolic differences between *E. coli* and *P. aeruginosa* make it ambiguous whether self-secreted heterologous PCA would be utilized in MES performed by *E. coli* and unknown are the effects it might have on growth and metabolism under the electrochemical tension.

In this study, we investigated how the introduction of the PCA pathway of *P. aeruginosa* into *E. coli* affects PCA production and viability. Furthermore, the heterologously expressed PCA pathway was employed to transfer electrons from the cathode to the microbial cells and achieve reduction of fumarate in bioelectrochemical reactors at negative cathode potential. Through the measurement of electrochemical parameters and fermentation products, the effect of the heterologously expressed PCA pathway on metabolism of *E. coli* and bioelectrocatalytic activity under the electrochemical tension was analyzed. Additionally, we found that adenosine triphosphate (ATP), as the “energy currency,” was stimulated by the electrical current through the cathode provided by the inward EET process utilizing the heterologously self-excreted PCA in *E. coli*.

## Materials and Methods

### Bacterial Strains, Plasmids and Plasmid Construction

The strains BA102 and PAO1 of *E. coli* and *P. aeruginosa*, respectively, were used in this work (Table 1). Plasmids and construction of plasmids are described in our previous study (Feng et al., 2018, 2020). Recombinant plasmid ptrc99a*-phzA1-G1* was transformed into *E. coli* BA102 to obtain a PCA-producing strain (designated as *E. coli-phz)*. The deletion strain *E. coli* BA102 *(ΔmenA)* was constructed as described previously using the method of the CRISPR-Cas9 System (Feng et al., 2020). Firstly, the competent cells of *E. coli* BA102 harboring pCas were prepared as described previously by Jiang et al. (2015). Then donor DNA of *menA* and plasmid pTargetF-C-*menA* were constructed through PCR using the corresponding primers listed in Table 1 and *E. coli* BA102 genome and pTargetF-C as the template, respectively. The pTargetT-*menA* (100 ng) and donor DNA (500 ng) were mixed and then added to a total of 50 μl of competent cells. Introduction of the pTargetT-*menA* and DNA into *E. coli* cells was carried out by electroporation at 2.5 kV. After being inoculated in 1 mL Luria-Bertani (LB) medium at 30°C for 1 h, cells were harvested by centrifugation (4000 rpm) for 3 min, spread onto LB agar plates and then incubated overnight at 30°C. 50 μg/ml kanamycin and 34 μg/mL chloramphenicol were added in LB agar plates for selection. Finally, transformants were identified by PCR using *men*A-confirm-1/*men*A-confirm-2 as primers and gene sequencing. The plasmid ptrc99a*-phzA1-G1* was transformed into *E. coli* BA102 *(ΔmenA)* to obtain *E. coli-phz (ΔmenA).*

**TABLE 1:** Strains, plasmids, and primers.

Strain/plasmid	Relevant characteristics	Reference/Source
**Bacterial strains**		
*E. coli* BA102	*Δldh,Δpfl,ΔptsG*	Lab collection
*P. aeruginosa*PAO1	Wild-type	Holloway et al. (1979)
**Plasmid**		
ptrc99a	Cloning Vector;Amp^*r*^	Takara Inc.
ptrc99a-*phzA1-G1*	ptrc99a carrying the *phzA1B1C1D1E1F1G1* gene cluster of *P. aeruginosa* PAO1 under constitutive *trc* control; Amp^*r*^	This work
pTargetF-C	pTarget series harboring sgRNAs-Cm	This work
pCas	repA101(Ts) kan Pcas-cas9 ParaB-Red lacIq Ptrc-sgRNA-pMB1	Jiang et al. (2015)
pTargetF-C-*menA*	pMB1 aadAsgRNA-menA	This work

**Primers**	**Oligonucleotides**	**Reference/Source**

*men*A-sgRNA-up	AATACTAGTGCTACGCGGCATGCAAAAAGGTTTTAGAGCTAGAAATAGC(N20)	This work
*men*A-sgRNA-down	CTTTTTGCATGCCGCGTAGCACTAGTATTATACCTAGGACTGAGC	This work
*men*A-Donor-1	AGCGTTTAATGGAAGAGATTTCCTACGAC	This work
*men*A-Donor-2	CGTTGCCAGCAGCTAATTTGTTGTTCAGTCATAATACGCG	This work
*men*A-Donor-3	ACAACAAATTAGCTGCTGGCAACGGCAG	This work
*men*A-Donor-4	CGATATACTGAAAATTCTCGCAGCAACTGAAT	This work
*men*A-confirm-1	CCTGCTGCCGCTGGTAGAAG	This work
*men*A-confirm-2	CCAACAGGTAACGCAGAAAAAAGGC	This work

*sgRNA-menA: sgRNA with an N20 sequence for targeting the menA locus.*

### Media and Growth Conditions

Samples of *E. coli* and *P. aeruginosa* stored at −80°C were pre-grown for 8–12 h at 37°C in LB medium. Then each culture suspension (1%) was used to inoculate100 mL LB medium and cultured at 37°C and 200 rpm. When *E. coli-phz* grew to OD_600_ about 0.6, an inducer (IPTG) was added for *phzA1-G1* expression. After an additional 10–12 h of growing, cultures were saved for further analysis and electrochemical measurements. For *E. coli-phz*, ampicillin (100 μg/mL) was added into its LB medium in order to maintain selection for cells with the plasmid ptrc99a*-phzA1-G1* in *E. coli*.

### Construction and Operation of Bioelectrochemical Reactor

A dual chamber bioelectrochemical reactor (internal volume per chamber of 250 ml) was constructed. The two reaction chambers were separated by a proton exchange membrane (Nafion, diameter, 35 mm, DuPont Co., United States). The cathode and anode electrodes were made of fine carbon felts (5 mm thick; 80 mm × 40 mm). The cathode was imposed at −0.6 V using an Ag/AgCl electrode as the reference electrode. The anode medium was made up of the following: 35.8 g⋅L^–1^ NaHPO_4_.12H_2_O, 15.6 g⋅L^–1^ NaH_2_PO_4_.2H_2_O, and 5.8 g⋅L^–1^NaCl, adjust to pH 7.2 with NaOH. We filtered dithiothreitol (DTT, 0.02%) through a 0.22 nm syringe filter and then added it into the anode medium before use. The cathode medium contained: 31.5 g⋅L^–1^ NaHPO_4_.12H_2_O, 8.5 g⋅L^–1^ NaH_2_PO_4_.2H_2_O, 10.0 g⋅L^–1^ NaHCO_3_, and 5.0 g⋅L^–1^ yeast extract. Prior to inoculation, sodium fumarate or D-glucose was added into the cathode medium and the pH of the cathode medium was adjusted to approximately 7.0 through flushing with CO_2_. The bacterial culture and cathode medium were mixed in a ratio of 1:2 and then inoculated into the cathode chamber. NaHCO_3_ and gaseous CO_2_ were added into bioelectrochemical reactors as carbon sources for MES. Bioelectrochemical reactors and serum bottles were stirred using a magnetic stirrer at 200 rpm and kept in incubators at 35 ± 1°C. The headspace of the cathode chamber inserted an airtight syringe to take samples regularly.

### Cell Growth and Quantification of ATP

The cell density was tracked via absorbance (OD_600nm_) by using a UV–visible spectroscopy system. The injection volume was 20 μl. A BacTiter-Glo^TM^ Microbial Cell Viability Assay (Promega, G8230, United States) was used to determine the concentrations of ATP.

### Organic Acid and PCA Analysis

The organic acid concentrations were assayed by an HPX-87H column (300 mm, −7.8 mm, Bio-Rad, Hercules, CA, United States) using a high-performance liquid chromatography (HPLC) system (Agilent Technologies, Santa Clara, CA, United States). HPLC was equipped with a refractive index detector set at 35°C and an ultraviolet spectrophotometric detector monitored at 215 nm. The column temperature was set to 60°C. The mobile phase was 8 mM sulfuric acid at a flow rate of 0.5 mL/min.

Phenazine-1-carboxylic acid was extracted from the culture supernatants according to a previously reported method (Feng et al., 2018) and analyzed by using HPLC system, which was equipped with an ultraviolet spectrophotometric detector set at 280 nm and a C18 analytical column (5 mm particle size, 250 mm, 4.6 mm i.d.) (Yong et al., 2011; Feng et al., 2018). The mobile phases were water with 0.05% acetic acid and acetonitrile with 0.05% acetic acid, respectively. The chromatographic separations utilized a linear gradient from 15% acetonitrile with 0.05% acetic acid to 83% within 20 min, followed by 15% acetonitrile with 0.05% acetic acid from 20 to 25 min at a flow rate of 1 mL/min.

### Electrochemical Analysis

Cyclic voltammogram (CV) measurements were performed by an electrochemical instrument (PMC 1000/DC, AMETEK, United States) in a three-electrode configuration using an Ag/AgCl electrode as the reference electrode. CV measurements were employed with a scan range of −800 to 600 mV and a scan rate of 20 mV/s.

Electrochemical impedance spectroscopy (EIS) was measured to the Nyquist plots of impedance on PMC 1000/DC and the frequency was decreased at an amplitude of 10 mV from 10^5^ to 0.01.

## Results and Discussion

### Successful Introduction of the Heterologous PCA Pathway Into *E. coli*

The engineered *E. coli-phz* was constructed by transforming ptrc99a*-phzA1-G1* into *E. coli* BA102. The parental strain *E. coli* BA102 was constructed by disabling the *pflB* gene, *ldhA* gene, and *ptsG* gene to eliminate main competing fermentation pathways and improve succinate production. The culture medium of *E. coli-phz* after 12 h of culturing appeared yellow (Supplementary Figure 1), resulting from the color of the pigment of the phenazine PCA. Similar to the data presented by our previous reports (Feng et al., 2018), a significant peak of PCA was found in the extracts of *E. coli-phz*, while no PCA synthesis was observed in *E. coli* BA102 (Supplementary Figure 2). These results reflect that the *phzA1-G1* gene from *P. aeruginosa* PAO1 was efficiently expressed and PCA was synthesized in *E. coli-phz*.

**FIGURE 1:**
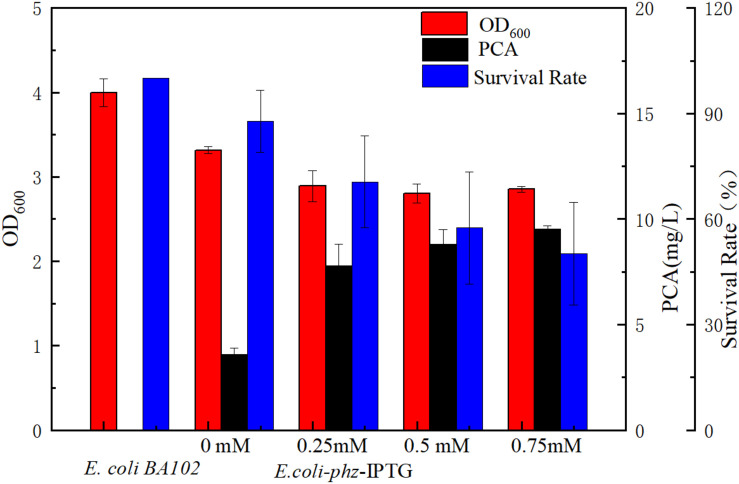
The effect of *phzA1-G1* gene expression on PCA production (black) and cell growth (red) at the aerobic growth stage and survival rate (blue) of cells in the bioelectrochemical system at 8 h. Cell survival rate shows the changes of OD_600nm_ at 8 h/OD_600nm_ at 0 h in the bioelectrochemical system compared with that of *E. coli*BA102.

To investigate the effect of heterologous PCA on cell growth, PCA production, and OD_600nm_ values were assessed by titration of various IPTG concentrations ranging from 0 to 0.75 mM. With increasing IPTG concentration, PCA production also gradually increased which demonstrated that PCA was synthesized to 3.87 mg/L in *E. coli* even relying on basal expression (Jensen et al., 2010). However, cell growth was gradually inhibited as shown in Figure 1. Of the *E. coli* BA102 and *E. coli-phz* strains separately inoculated into the bioelectrochemical reactor and imposed at −0.6 V (vs. Ag/AgCl), both presented decreases of cell number in the first 8 h. The survival rate of *E. coli-phz* decreased from 87.86 to 57.51% as the IPTG concentrations increased from 0 to 0.5 mM in the bioelectrochemical system (Figure 1). The results indicate that the electrical current might have led to cellular damage. To ensure the presence of a sufficient amount of living cells, all subsequent experiments were performed in the absence of IPTG.

### The Function of Self-Excreted PCA as the Endogenous Electron Mediator in the Bioelectrochemical System Driven by Electricity

To test the function of the endogenous electron shuttle PCA in an bioelectrochemical system, the reaction of fumarate reduction to succinate was carried out, which is a simple one-step reaction with electrons transfer and an essential catalyzed reaction for microbes (Fang et al., 2020). The self-excreted PCA did not show significantly high efficiency. Some exogenous electron mediators, such as neutral red, 2,6-dichloroindophenol, potassium ferricyanide, and Lauth’s violet also showed low efficiency in MES by *E. coli* according to previous report (Wu et al., 2019). In the bioelectrochemical systems, cellular damage caused by the electrical current led to a reduced survival rate of *E. coli-phz* (Figure 1). Considering the difference in cell density between *E. coli-phz* and *E. coli* BA102, the catalytic effect of per unit *E. coli-phz* cells (succinate concentration/OD_600_) was investigated. As the results illustrate in Table 2, the succinate/OD_600_ of *E. coli-phz* was up to 137%, compared with that of *E. coli* BA102 cells. The ability of PCA to transfer electrons likely promoted the succinate production of per unit *E. coli-phz* cells. We conclude that self-excreted PCA could transfer electrons between the cell and the electrode by introducing the heterologous PCA pathway into *E. coli*.

**TABLE 2:** Fermentation profiles in bioelectrochemical systems of *E. coli* BA102 and *E. coli-phz*.

Strains	Succinate* (g/L)	Succinate (g/L)	Acetate (g/L)	Pyruvate (g/L)	Initial OD_600nm_	Terminated OD_600nm_
*E. coli* BA102	3.85 ± 0.20	6.93 ± 0.26	1.57 ± 0.52	2.88 ± 0.33	1.05 ± 0.10	1.53 ± 0.31
*E. coli-phz*	5.26 ± 0.38	7.26 ± 0.22	1.21 ± 0.23	1.98 ± 0.28	0.90 ± 0.02	1.01 ± 0.13

Succinate*: succinate concentration/OD_600_ with fumarate as the substrate.

### Heterologous PCA Pathway Enhanced Bioelectrocatalytic Activity

**FIGURE 2:**
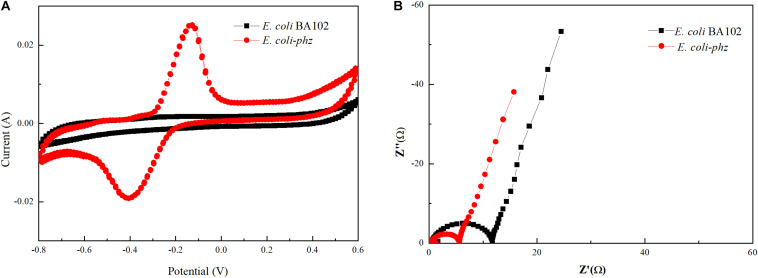
Effects of self-excreted PCA on bioelectrocatalytic activity in bioelectrochemical system. Cyclic voltammetry **(A)** and Nyquist plots **(B)**. *E. coli* BA102 (black square) and *E. coli-phz* (red circle).

In order to elucidate the effect of the heterologous PCA pathway on bioelectrocatalytic activity, CV and EIS measurements were obtained. CV was used to investigate the redox reactions at the microbe–electrode interface to reveal the characterization of the bioelectrocatalysis (Liu et al., 2005). The redox species that took part in EET in the electrochemical systems were determined by CV analysis. The CV data indicated that there was no significant redox peak in the bioelectrochemical system inoculated with *E. coli* BA102 (Figure 2A), which showed that there was no significant redox reactions between *E. coli* BA102 cells and electrode. In contrast to *E. coli* BA102, a significant pair of redox peak was observed from the bioelectrochemical system inoculated with *E. coli-phz.* Figure 2A showed an oxidation peak in the range of 0.0 to −0.20 V and reduction peak between −0.30 and −0.50 V, which agreed with the broad PCA redox peak system determined for *P. aeruginosa* PA14 (Bosire et al., 2016). The results indicate that self-excreted PCA functioned as electron mediator in the bioelectrochemical system inoculated with *E. coli*-*phz*, which achieved the utilization of electrons and redox reaction in the bioelectrochemical system inoculated with *E. coli*-*phz.*

Nyquist plots of impedance obtained by EIS measurements can precisely evaluate the electron transfer resistance of *E. coli-phz* and *E. coli* BA102 (He and Mansfeld, 2009; Nandy et al., 2016). The diameter of the well-defined semicircle curve in the high frequency region was equivalent to the electron transfer resistance. As shown in Figure 2B, the charge transfer resistance of the *E. coli-phz* system was lower than that of *E. coli* BA102 system, indicating there was a higher electron transfer rate in *E. coli-phz*. These results demonstrate that self-excreted PCA in *E. coli-phz*, accomplished by introducing the heterologous PCA pathway in *E. coli*, was the main electron mediator employed in electron transfer reactions that improved the EET between microbial cells and the electrode.

### Heterologous PCA Pathway Affected the Central Metabolism With Glucose as the Carbon Source in Bioelectrochemical System

In previous experiments, researchers have successfully increased conversion of succinate from fumarate in MES (Harrington et al., 2015b; Wu et al., 2019; Fang et al., 2020). Microbial electrosynthesis offers a possible route to move beyond the limitation of metabolic redox state and disturb intracellular metabolism. To better understand how the heterologous PCA pathway can alter cell growth and a metabolism driven by electrical current, glucose (final concentration of 10 g/L) was used as the carbon source. Fermentation products, including succinate, pyruvate and acetate were measured by HPLC to investigate changes in cellular metabolism. Succinate was produced at about 7.26 g/L by *E. coli-phz* and 6.93 g/L by *E. coli* BA102 after 45 h in culture (Table 2). The final optical density (OD_600_) of *E. coli-phz* was lower than that of *E. coli* BA102, which suggested that cell growth of *E. coli-phz* was inhibited compared to the increasing cell growth of *E. coli* BA102. This demonstrates that the production capacity of succinate per unit of *E. coli-phz* was higher than that of *E. coli* BA102. The higher titer of the reduced production was in good agreement with the result of MES by *E. coli* with NR as exogenous electron mediator, which was due to the utilization of electricity (Harrington et al., 2015a; Wu et al., 2019). Since PCA was self-excreted by *E. coli-phz* in this work, the problems on exogenous addition and the cost were dealt with.

In addition, because of the *pflB* deletion, pyruvate accumulated as one of main by by-products. As shown in Table 2, *E. coli-phz* displayed a greater ratio of succinate/pyruvate and succinate/acetate contents than those of *E. coli* BA102. The results demonstrate that self-secreted PCA of *E. coli-phz* could transfer electrons from a cathode to intracellular electron carriers. Furthermore, microbial cells were stimulated and changed the intracellular metabolite profiles to facilitate the production of reduced metabolites driven by electrical current.

### The Limiting Factor of *E. coli* Cells in Bioelectrochemical System

**FIGURE 3:**
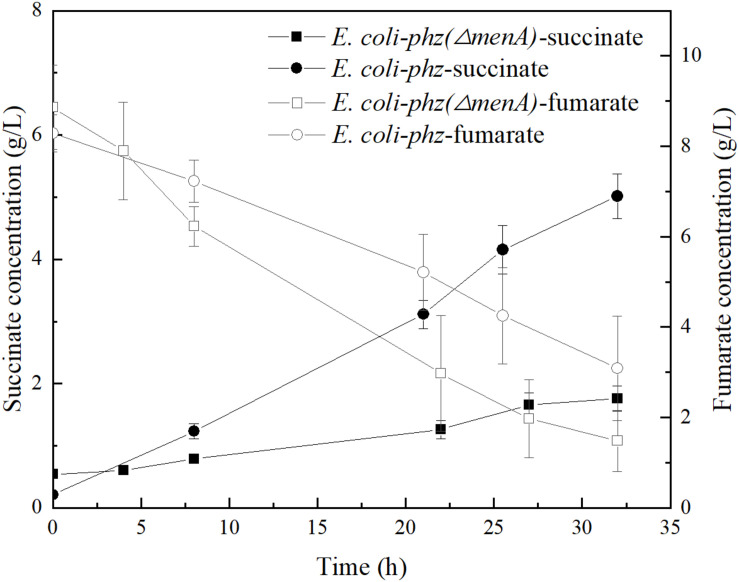
The concentrations of succinate and fumarate in the bioelectrochemical systems of *E. coli-phz* (black circle) and *E. coli-phz(ΔmenA)* (black square).

It is well known that microbial cells in bioelectrochemical system (MES and EF) interact with the electrode through a series of oxidation/reduction and electron transfer reactions to drive the biocatalysis reactions or supply electrons from the cathode. In addition, some research have shown that intracellular energy metabolism, biological activity and biofilm formation can be stimulated and changed by providing electrical currents through cathodes (Yong et al., 2011). In our previous experiments, we observed retarded cell-growth in a bioelectrochemical system inoculated with *E. coli-phz* compared to the inoculated with *E. coli* BA102. According to previous literature reports, the growth of *P. aeruginosa* was inhibited by electrical stimulation (both cathodal and anodal stimulation) (Kincaid and Lavoie, 1989; Szuminsky et al., 1994; Asadi and Torkaman, 2014). In bioelectrochemical system, the cell growth or biomass generation is related to the molecular mechanisms of ET from the cathode to terminal electron acceptor in the microbes (Harrington et al., 2015b). As Clark and Harrington et al. have indicated, the major reaction in the inner membrane of *E. coli* is menaquinone (MK) reduction in mediated bioelectrochemical system (Clark et al., 2012; Harrington et al., 2015b). In NR-mediated bioelectrochemical system, NRH_2_ gains electrons from the cathode and diffuses into the inner membrane, where MK is reduced to MKH_2_ by NRH_2._ MKH_2_ then passes electrons to the terminal reductase with concomitant reduction of the electron acceptor to reduced end-product (Harrington et al., 2015b). In order to test the role that MK play in self-excreted PCA-mediated electrosynthesis, the *menA* deletion strain *E. coli* BA102 *(ΔmenA)* was constructed and ptrc99a*-phzA1-G1* was transformed into *E. coli* BA102 *(ΔmenA)* to obtain *E. coli-phz (ΔmenA).* Fumarate was added to the bioelectrochemical system culture of *E. coli-phz (ΔmenA)* to compare the consumption of fumarate and the accumulation of succinate with *E. coli-phz*, respectively. As shown in Figure 3, disabling the *menA* gene resulted in the normal consumption of fumarate but significantly lower accumulation of succinate in MES system. This suggests that MK was critical to the reduction of fumarate to succinate in bioelectrochemical system of *E. coli*. The first relay station of fumarate reductase to use exchange electrons with quinines has been confirmed to be the center3[3Fe-4S] cluster of fumarate reductase (Heering et al., 1997). Given these results, MK is one of the key components in PCA-mediated electrosynthesis.

**FIGURE 4:**
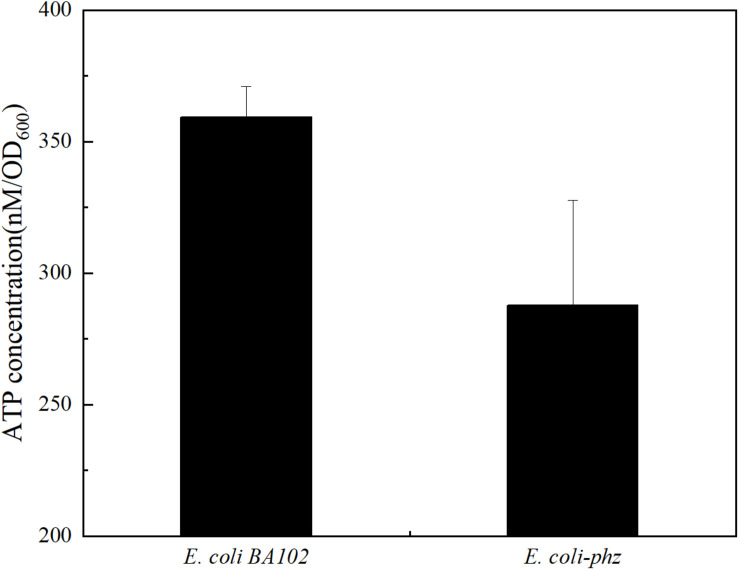
The ATP levels of *E. coli* BA102 and *E. coli-phz*.

According to the hypothesis, electrons of MKH_2_ are derived from reduced PCA instead of NADH via NADH dehydrogenase (Complex I) partly, resulting in lower ATP yields and growth inhibition. The reaction that MK is reduced by NADH via Complex I can contribute the proton motive force, which is used to drive ATP synthase to synthesize ATP. If reductant is PCA, the proton motive force was not generated. ATP is often referred to as the “energy currency” in all living microbial cells that supplies energy for cellular functions and processes. It is also useful in research as an independent bio-indicator of microbial metabolic viability and activity. To test the hypothesis, we measured ATP contents and observed a lower level of ATP in *E. coli-phz* than *E. coli* BA102 in bioelectrochemical systems (Figure 4). To further verify the effects of ATP on the biomass and the production of succinate, ATP was exogenously added in the bioelectrochemical system inoculated with *E. coli*-*phz.* The results showed that the terminated OD_600_ of *E. coli-phz* increased by 19.4% by the exogenous addition of ATP, but there was not an increase in succinate production compared with that of the control group without addition of ATP (Supplementary Figure 3). The results suggested that there were other factors affect biomass generation and succinate production, such as instable chemical property of ATP, the low external redox-potential environment (Wu et al., 2019), intracellular unbalanced redox state (Heux et al., 2006; Liang et al., 2013) induced by the transfer and utilization of the cathode electrons. To achieve more efficient electrosynthesis, screening of the electricity-tolerance strain would be carried out and the electrosynthesis conditions would be further optimized.

## Conclusion

*Escherichia coli* BA102 can generate and excrete PCA after being transformed to express *phzA1B1C1D1E1F1G1* from *P. aeruginosa* PAO1. Engineered *E. coli* in bioelectrochemical system using self-excreted PCA as electron transfer mediator allowed electrons on the cathode to be transferred to the inner membrane, leading to the connection between the electrode and microbial cells and improvement of electron transfer efficiency. Driven by the electrical current, the influence of a heterologous electron transfer mediator on cell growth, fermentation production, and bioelectrocatalytic activity of *E. coli* cells was studied. In the self-excreted PCA-mediated bioelectrochemical system, the electrical current led to cellular damage and affected cell growth. However, succinate production increased and by-products declined. The results demonstrated that the electrical current of *E. coli-phz* altered the metabolism to facilitate the production of reduced metabolites. Meanwhile, synthesis of ATP, the “energy currency,” was stimulated by the electrical current through the cathode. It also found that MK was critical to the reduction of fumarate to succinate in bioelectrochemical system of *E. coli*. This study highlights the ability to transfer electrons between electrode and microbial cells by engineering bacteria to produce self-excreted electronic mediators to facilitate bioproduction that is driven by bioelectrical currents. It provides ideas for the application of heterologous electron transfer mediator excreted by *E. coli* in MES or EF and lays a foundation for subsequent optimization.

## Data Availability Statement

The original contributions presented in the study are included in the article/Supplementary Material, further inquiries can be directed to the corresponding author.

## Author Contributions

JF and KC designed the project. JF, KL, and QL conducted and analyzed the experiments. SX provided technical assistance. JF analyzed the data and wrote the manuscript with input from SX, XW, KC, and PO. All authors reviewed and approved the manuscript.

## Conflict of Interest

The authors declare that the research was conducted in the absence of any commercial or financial relationships that could be construed as a potential conflict of interest.
