# Supplementary material for: Construction of an Electron Transfer Mediator Pathway for Bioelectrosynthesis by Escherichia coli
Source: Front Bioeng Biotechnol. 2020 Oct 15;8:590667. doi: 10.3389/fbioe.2020.590667 (PMC7594510; doi:10.3389/fbioe.2020.590667)
Supplement: Supplementary file 1 [file Data_Sheet_1.PDF]

Supplementary Material

Supplementary Figures

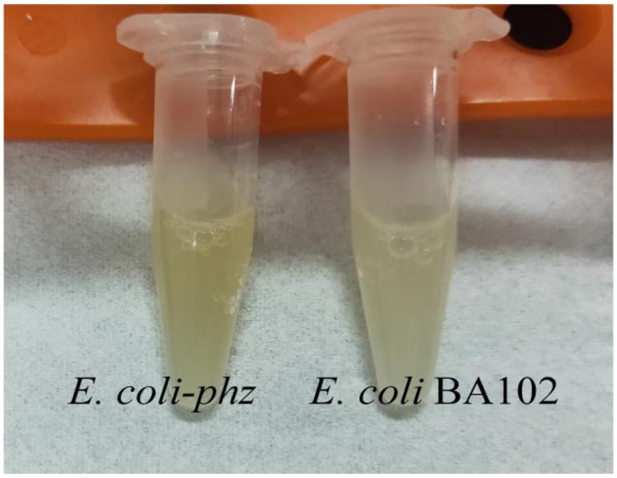

Supplementary Figure 1. Phenazines pigment production by *E. coli* BA102 and *E. coli-phz* under uninduced conditions

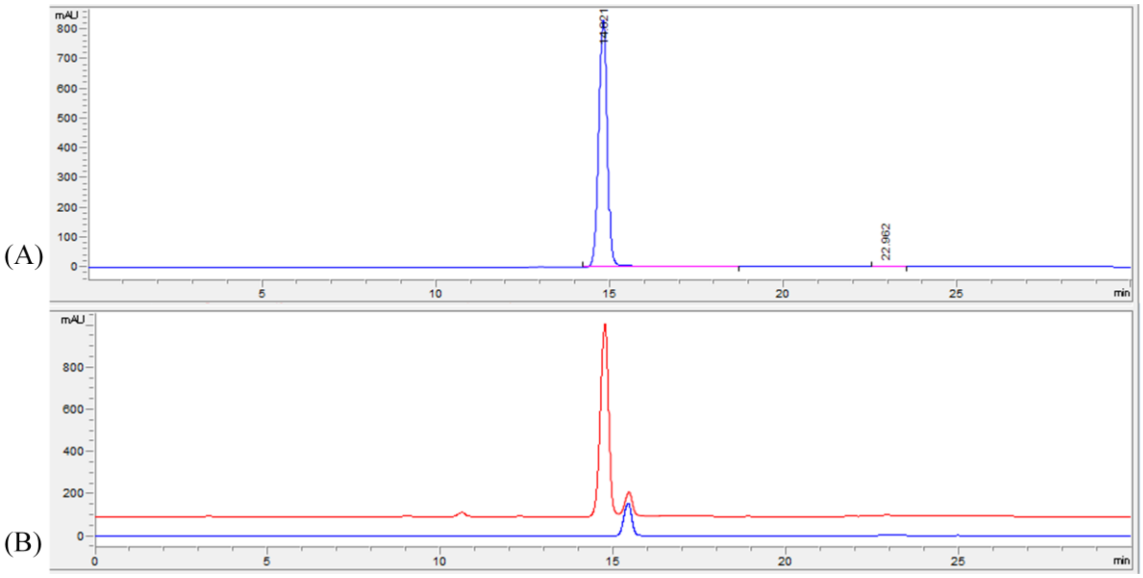

Supplementary Figure 2. The HPLC chromatogram of PCA (A); The HPLC chromatogram of the supernatants for *E. coli* BA102 (blue) and *E. coli-phz* (red) (B).

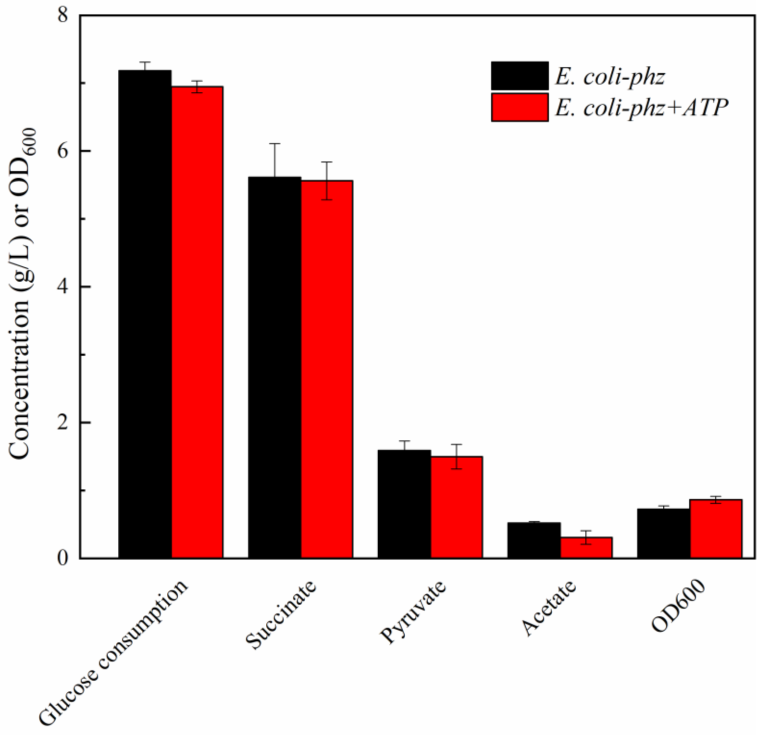

Supplementary Figure 3. Fermentation profiles in bioelectrochemical systems of *E. coli-phz* with (red) or without(black) ATP at 35 h.
